# Periodontal regeneration: Lessons from the periodontal ligament-cementum junction in diverse animal models

**DOI:** 10.3389/fdmed.2023.1124968

**Published:** 2023-03-07

**Authors:** Eli D. Sone, Christopher A. McCulloch

**Affiliations:** ^1^ Institute of Biomedical Engineering, University of Toronto, Toronto, ON, Canada; ^2^ Department of Materials Science and Engineering, University of Toronto, Toronto, ON, Canada; ^3^ Faculty of Dentistry, University of Toronto, Toronto, ON, Canada

**Keywords:** cementogenesis, mineralization, periodontitis, Sharpey’s fibers, cementoblasts

## Abstract

The attachment of the roots of mammalian teeth of limited eruption to the jawbone is reliant in part on the mineralization of collagen fibrils of the periodontal ligament (PDL) at their entry into bone and cementum as Sharpey's fibers. In periodontitis, a high prevalence infection of periodontal tissues, the attachment apparatus of PDL to the tooth root is progressively destroyed. Despite the pervasiveness of periodontitis and its attendant healthcare costs, and regardless of decades of research into various possible treatments, reliable restoration of periodontal attachment after surgery is not achievable. Notably, treatment outcomes in animal studies have often demonstrated more positive regenerative outcomes than in human clinical studies. Conceivably, defining how species diversity affects cementogenesis and cementum/PDL regeneration could be instructive for informing novel and more efficacious treatment strategies. Here we briefly review differences in cementum and PDL attachment in commonly used animal models to consider how species differences may lead to enhanced regenerative outcomes.

## Introduction

Mammalian teeth of limited eruption are attached to the jawbone through the exquisitely controlled mineralization of collagen fibrils of the periodontal ligament (PDL) at their entry into bone and cementum as Sharpey's fibers. The unusually abrupt interface of mineralization at the PDL-bone and PDL-cementum junctions requires high precision spatial regulation of fundamental biological processes that are not well understood. On the surface of the tooth root, cementum is the thin, mineralized connective tissue of mammalian teeth into which PDL fibres are anchored in the cervical portion of the root (1). This form of tooth attachment, known as a “true” gomphosis, is unique to mammals; in many other vertebrate species, teeth are rigidly attached to the jawbone through ankylosis (2). The maintenance of an unmineralized PDL that is robustly anchored to the tooth root and to the jawbone, provides stable anchorage along with stress absorption and sensory input that regulates masticatory muscle activity. This system also permits physiological tooth drift or the translational movement that occurs in orthodontic treatment *via* remodeling of the PDL-bone interface (3).

Periodontitis is a high prevalence infection of periodontal tissues in which the attachment of the PDL to cementum is destroyed by host proteases; without treatment, destruction of bone and PDL can ultimately lead to tooth loss. One of the goals of regenerative periodontal therapies is to restore tissues destroyed by periodontitis, including bone, cementum, and the fibrous attachments of the PDL and gingiva, along with reformation of the epithelial components of the dentogingival junction (4). The focus of this article is the formation of a functional PDL-cementum interface in which PDL fibers are inserted into the cementum orthogonal to the root, which remains a significant limiting factor in periodontal regeneration. Despite the high prevalence of periodontitis and many decades of research into various possible treatments, reliable restoration of periodontal attachment after surgery is still not achievable. Clinical (5) and animal (6) studies show that while regeneration can occur, the amount of regeneration achieved is limited and unpredictable (7). Accordingly, there is a critical need for improved understanding of the regulation of cementogenesis after periodontal treatment. Most notably, we need to define what regulates the mineralization of oriented PDL fibers at the cementum junction if we are to develop effective strategies for achieving *de novo* periodontal attachment. In this review we consider the potential inputs of cellular, molecular, and mechanical regulators into these processes. Further, as periodontal regeneration involves many different cell populations, more types of tissues and complex regulatory systems than the attachment of endosteal implants to bone, we need to consider how these differences should inform our formulation of objectives and hypothesis testing.

Notably, human clinical studies using alloplastic materials have often reported unexceptional regenerative outcomes compared with animal studies that used similar materials and reported markedly positive results (8). This is particularly relevant with respect to the reformation of a functional PDL-cementum attachment following attempts to regenerate periodontium following periodontitis in humans. Further, these differences suggest that a consideration of species diversity in cementogenesis and cementum/PDL regeneration may be instructive for informing novel and efficacious strategies to improve clinical outcomes. Here we review differences in cementum and PDL attachment in development, in mature tissues, and in regeneration. Throughout the text, the following questions are explored:

(i) *Why are the outcomes following periodontal regenerative procedures in humans much less compelling than animal models?* Here we consider that limitations in the models, and in the still relatively-poorly developed knowledge of human periodontal regeneration, influence the notion that human periodontitis lesions are intractable in terms of regeneration.
(ii) *Which cells are involved in cementum/PDL regeneration*? In spite of a large volume of research, fundamental questions remain regarding the lineages of progenitor cells involved in the regeneration of these tissues, and what factors affect the differentiation of these cell types. Compared with the wealth of knowledge about progenitor cells of tissues such as skin, blood, and small intestine, this field is ripe for further investigation.
(iii) *What signals (soluble molecules, extracellular matrix, mechanical factors) direct local cells to form cementum/PDL?* The formation of the PDL-cementum interface relies on spatial and temporal coordination of several complex cellular processes. For rational design of regenerative strategies and materials, it will be important to understand which signals control gene expression (and therefore, cell behavior) to improve regenerative outcomes.

## Cementogenesis and PDL attachment in developing periodontium

In lesions of periodontal tissues such as periodontitis, inserting collagen fibers and periodontal ligament cells are depleted from the cementum surface at affected sites, which is a central challenge for attempts to promote periodontal healing. Despite heroic efforts to repopulate denuded cementum surfaces with therapies such as guided tissue regeneration, topical application of bone morphogenetic proteins or the utilization of resorbable membranes, these approaches are associated with clinical outcomes that are either not predictable or do not completely restore the structure of the original periodontal tissues. For obtaining more substantial outcomes following repair of lost periodontal tissues in humans (i.e., that routinely exceed 3 mm of net attachment gain), we need to recapitulate those critical processes seen in tooth development. In particular, these processes include the promotion of cementogenesis and the insertion of Sharpey's fibers that are contiguous with the principal fibers of the PDL (9).

Arising from the recognition that the formation of cementum is an essential step for periodontal regeneration, substantial efforts have been devoted to identifying the origin of cementoblasts and the differentiation steps that lead to the generation of cementoblast precursors. Cementoblasts synthesize the various types of cementum that have been identified in teeth of limited eruption in mammals. While earlier data indicated that ectomesenchymal cells in the dental follicle can differentiate into cementoblasts during root development, other data supported the notion that cementoblasts originate from Hertwig's epithelial root sheath, which is thought to play a central role in directing root formation and cementogenesis (10) While little detailed knowledge is available on the cell lineages of cementoblasts in large animals and in humans, studies of regulatory systems in mice that impact cementogenesis have identified a broad array of signaling systems. These signaling systems include such examples as the Wnt/β-catenin and the Wnt non-canonical pathways (11) and Osterix (12). Future work that is oriented towards therapeutic control of cementogenesis as part of periodontal regeneration may need to identify the relative size, location, phenotypic repertoire and differentiation potential of cementoblast precursor cells in mice and larger animals, and to estimate the relative proportion of these precursor cells that can be attracted into healings wounds in order to repopulate the denuded cementum in treatments for periodontitis.

As many studies of cementogenesis have used mouse models to study fundamental processes that enable the formation of cementum, it is notable that similar to humans, there is very limited remodeling of cementum in adult mice. This feature of mouse periodontal tissues suggests that even though there are marked differences in many structural aspects of human and mouse periodontium, the common manifestation of restricted cementogenesis across mammalian species, indicates that the mouse could indeed serve as a useful model for more translational aspects of human cementogenesis. Notably, there are also important differences in cementogenesis between humans and rodents (13). For instance, in human premolars, the formation of acellular extrinsic fiber cementum (which is involved in tooth attachment) occurs on previously unmineralized dentin in such a manner that there is interdigitation of collagen fibers at the cementum-dentin junction prior to mineralization. In rodents, the dentin surface is already mineralized prior to the deposition of acellular extrinsic fiber cementum. Further, the first molar tooth of small rodents is in function within weeks of the onset of root formation, while in human premolars a much longer time (years) is required for root development and initial stages of cementogenesis prior to occlusal loading. Nevertheless, in spite of the very large evolutionary distance between mice and humans, and between more evolved mammals and humans, we can learn from these evolutionary differences to obtain better insights into how cementogenesis is regulated.

Ripamonti and colleagues have examined lower primates (14) and sharks. They have made interesting observations on whether species-dependent diversities of tooth morphogenesis and root development can be instructive for periodontal tissue regeneration and in particular, cementogenesis (15). In this research, they produced data to show that TGF-β3 can promote cementogenesis in lower primates, although the nature of the target population(s) and their developmental repertoires are not defined. In this context, critical pieces of information may ultimately be obtained from spatial transcriptomics data of mRNA repertoires in order to provide a broader understanding of the range of phenotypes that are manifest in the differentiation of cementoblast precursor cells (16). The potential differentiation repertoires exhibited by cementoblast precursors and their presumably more differentiated progeny, and how these repertoires may differ between developing and mature root surfaces, are likely to inform rational development of therapies that can effect cementogenesis in the fully erupted teeth of humans affected by periodontitis.

An important difference between healthy but surgically reduced periodontal tissues in animal models (i.e., periodontal tissue removed surgically but in the absence of periodontitis) and human periodontitis lesions is that poorly quantified and understood alterations to the chemistry of cementum surfaces affected by periodontitis are difficult to compare with surgically reduced periodontal tissues in the animal models. These differences could strongly impact the outcomes of experimental surgical interventions that are used to effect tissue regeneration. Frequently, surgically reduced animal models exhibit impressive gains in cementum formation after experimental therapies that are not easily demonstrable after treatment of periodontitis in humans. Accordingly, the use of proteomic methods (17) to quantify the complex repertoire of proteins in human cementum would help to advance the field of regeneration by pinpointing critical differences between diseased and healthy cementum.

## Control of mineralization at the PDL-cementum interface

**Figure 1:**
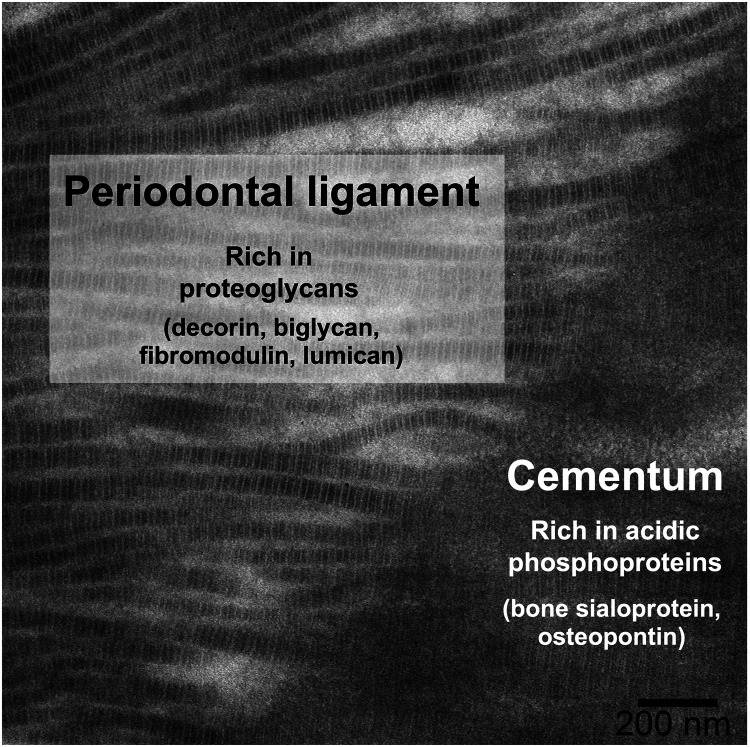
Distribution of some key non-collagenous macromolecules at the PDL-cementum junction, shown on a TEM micrograph of a demineralized mouse mandibular molar root, in which insertion of PDL collagen fibrils into the cementum can be observed.

The extracellular matrix of acellular cementum is composed primarily of Type I collagen, with smaller amounts of Type III collagen and non-collagenous macromolecules (1). The collagen fibrils of cementum are mineralized with hydroxyapatite, similar to dentin and bone, while PDL fibrils are unmineralized. Several non-collagenous macromolecules, chiefly proteoglycans and phosphoproteins, are thought to control aspects of mineral formation (inhibition vs. nucleation) and growth (18, 19). The distribution of proteoglycans (e.g., decorin, biglycan, fibromodulin, and lumican) (20) and phosphoproteins (e.g., bone sialoprotein and osteopontin) (21) at the periodontal ligament–cementum junction (Figure 1) provides clues of their roles in control of mineralization: initiation of mineralization is thought to be related to loss of ligament proteoglycans (22, 23) and/or the presence of acidic phosphoproteins in cementum (24, 25). But neither of these hypotheses has been fully validated because: (i) many of these proteins have multiple and/or redundant roles, which complicates interpretation of knockout models (26); and (ii) there are few *in vitro* models that enable untangling the many variables separately while reproducing the complex tissue environment (27–30). In this context, we have developed an *in vitro* model of mineralization based on remineralization of demineralized sections of mouse periodontium, in which we demonstrated that the extracellular matrix contains sufficient information to control the rate of mineralization into mineralized tissues (bone, cementum, dentin) as compared to the non-mineralized PDL (31). We further used selective enzymatic digestions to explore the respective roles in mineralization of phosphoproteins (32) and glycosaminoglycans (GAGs) (33), the linear polysaccharides often attached to a protein core in proteoglycans. This latter research demonstrated that GAGs play an important role in promoting mineralization in dental tissues rather than inhibiting mineralization in the ligament.

A comparison of the distribution of BSP and OPN in human premolars and rat molars revealed a similar pattern between the two species, with differences in the timing of their appearance consistent with developmental differences reflected in the rate of tooth eruption (24). A more comprehensive review of the proteins present in cementum has been provided earlier (34), and recently a comparative proteomic analysis of cementum in human deciduous and permanent teeth was conducted, with clear differences identified in their proteomes (35). With respect to GAGs, species differences have been identified in their distribution in cementum and PDL, as summarized in Table 1. Chondroitin sulphate is a major GAG in cementum and PDL and is found across different species. Dermatan sulphate, keratan sulphate, and hyaluronan are present only in specific tissues and exhibit wide species-dependent variations. While the impact of species differences on the distribution of these GAGs is poorly described, we note that even if the same type of GAG is present, there may be differences in chain length and/or sulphation level. These differences can affect functions such as modulation of collagen fibrillogenesis (44) and binding of hydroxyapatite (45). A better understanding of the manner in which non-collagenous macromolecules can influence mineralization may help in the design of scaffolds for recreating the critical PDL-cementum interface in periodontal regeneration.

**Table 1:** Distribution of GAGs in dental tissues [modified from Wojtas et al. (33)].

Tissue	Organism	CS	DS	KS	HA
Cementum	Human (36)	++	−	−	+
	Cow (37)	+	n/d	n/d	n/d
	Rat (38)	+	−	−	n/d
	Mouse (39)	+	−	n/d	n/d
	Sheep (40)	++	+	n/d	−
PDL	Cow (41)	++	n/d	n/d	+
	Rat (42, 43)	+	n/d	+	+
	Mouse (39)	+	++	n/d	n/d
	Sheep (40)	+	++	n/d	+

n/d - not determined;+present; ++ most abundant; - not present.

CS, chondroitin sulphate; DS, dermatan sulphate; KS, keratan sulphate; HA, hyaluronan.

Cellular regulation of mineralization in the periodontium has recently been reviewed by Foster and colleagues (46). The key effectors include regulators of phosphate metabolism, pyrophosphate metabolism, and extracellular matrix proteins as discussed above. Additional signaling pathways regulate mineralization indirectly by regulating the aforementioned factors, including TGFß, bone morphogenetic proteins, and Wnt-ß-Catenin signaling. The large majority of these studies were conducted in mice. The ability to use transgenic mouse models is a substantial advantage compared with other species as mouse models provide useful insights into human disease even though the possibility of species differences must be borne in mind. Several recent studies in mice have demonstrated the potential for new regenerative therapies based on knowledge of the regulators of cementum mineralization (47–49).

## Cementum and PDL regeneration in adult mammals

Investigations on the regenerative potential of the periodontium in a broad array of mammalian models have consistently focused on the primacy of the PDL and its resident cell populations in enabling repair and regeneration (50). This interest has arisen in part from studies of mice. Because of their small size, genetic homogeneity, refined definitions of the genetics of cell lineages, ease of experimentation and cost, mice have enabled deep insights into cell lineages in the PDL. For example, the newly identified periodontal ligament-associated protein-1 (Plap-1) in mouse is an extracellular matrix protein that may mark PDL-specific cell lineages. Recent data that focused on Plap-1 and how it might be used to identify progenitors of osteoblasts and cementoblasts, used RNA velocity analysis to show that Plap-1 positive cells contributed to PDL cell populations in normal tissues that contributed to periodontal regeneration (51). It is not known whether these same cell lineages contribute to periodontal regeneration in other species. It is also not clear how important any single protein is for the creation of periodontal cell lineages. A recent paper showed that Axin2-expressing cells in the PDL are needed for the development of the periodontium and that these cell populations are influenced by bone morphogenetic protein signalling (52). A separate study in mice recently identified two different stem cell populations that give rise to cementoblasts in homeostasis vs. disease (53), thereby advancing our understanding of cementoblast lineages.

An important advantage of using mice for studies of periodontal regeneration is the ability to conduct in-depth, cell lineage tracing, regulatory, and genetic studies of those critical elements that impact periodontal wound healing. But the extrapolation of data from mouse models for translation to the clinical management of the human periodontal lesion is fraught. We should remember the large size differences between human and experimentally-induced mouse lesions, the shape and structural differences between mouse and human teeth and root structures (particularly cementum) and periodontal ligament organization, and the challenges associated with generating authentic mouse periodontitis lesions. In In view of these limitations, there has been long-standing and extensive use of larger animal models including pig, sheep and dog.

Porcine models, similar to non-human primates such as Cynomolgus monkeys, exhibit marked similarities of their periodontal tissues to humans. They have thus been extensively used for studies of bone and periodontal regeneration (54). Dog models have also been used in studies of periodontal regeneration for over 50 years while sheep models have been employed much less extensively. While the size and similarities of cementogenesis in large animals are possible advantages for modeling of human cementogenesis, as reviewed recently (55), other factors in experimental models need to be considered as well. The dental and jaw anatomy and physiology of these animals, inter-animal variability, subtle differences of the morphology of tooth and root shape, and peculiarities of the structure of the periodontal ligament, have restricted our ability to obtain meaningful insights into regenerative responses compared with humans. This is particularly important for how certain biomaterials or cell transplantation methods could be optimized for translational purposes.

Large animal models are of particular value for cell-based tissue engineering and for optimizing pre-clinical, growth factor and cell-based treatments. Recent work using the non-human primate Chacma baboon, *Papio ursinus*, has underlined the processes that underpin periodontal tissue induction and regeneration using transforming growth factor-β (TGF-β) family proteins. The use of this non-human primate is a good example of how large animal models can advance our understanding of periodontal regeneration in general, and the peculiarities of cementogenesis in particular. There are some sites in the human jaws and dentition where it is difficult to obtain periodontal repair, let alone regeneration. This limitation is commonly seen in the furcations of molar and certain premolar teeth. Cognizant of these limitations, experiments have been conducted that use TGF-β family proteins to contribute to tissue morphogenesis and to the formation of functionally oriented periodontal ligament fibers that insert into nascent cementum. Notably, application of TGF-β3 in Matrigel® can promote the formation of new cementum in class II mandibular furcation defects previously created by surgical excision of tissue. In the newly created PDL space, collagen fibers are inserted into the cementum that forms on the dentine surface (15). This is a good example of the site-specific vagaries of achieving periodontal regeneration and how an unusually large animal model system may be of specific value for understanding basic processes that enable cementogenesis in sites that don’t normally heal.

## Conclusions

Diverse animal models have been used to study the periodontal ligament-cementum junction. These models include mice, pigs, sheep, dogs and non-human primates. The development and regeneration of the tightly controlled hard-soft tissue PDL/cementum interface in these animals often shares similarities to humans. Some of these animal models (e.g., transgenic mice) offer important advantages. Yet there are important differences in periodontal structure, molecular composition, and inducible disease state in these models compared with humans, which we considered here. Currently we do not know what role these differences play in enabling improved regenerative outcomes. This knowledge gaps arises in part because of fundamental, unanswered questions about cells, molecules, and mechanical signals that coordinate the formation of tooth attachment. Improved understanding of these processes, and of the differences between animal models and humans, will be instructive in informing progress in future periodontal therapies. Recent innovations in proteomics and spatial transcriptomics in particular bode well for advancing the field.
